# Treatment of lower urinary tract symptoms in men in primary care using a conservative intervention: cluster randomised controlled trial

**DOI:** 10.1136/bmj-2023-075219

**Published:** 2023-11-15

**Authors:** Marcus J Drake, Jo Worthington, Jessica Frost, Emily Sanderson, Madeleine Cochrane, Nikki Cotterill, Mandy Fader, Lucy McGeagh, Hashim Hashim, Margaret Macaulay, Jonathan Rees, Luke A Robles, Gordon Taylor, Jodi Taylor, Matthew J Ridd, Stephanie J MacNeill, Sian Noble, J Athene Lane

**Affiliations:** 1 Department of Surgery and Cancer, Faculty of Medicine, Imperial College, Hammersmith Hospital, London, UK; 2 Bristol Trials Centre, Population Health Sciences, Bristol Medical School, University of Bristol, Bristol, UK; 3 Department of Nursing and Midwifery, Faculty of Health and Applied Sciences, University of the West of England, Bristol, UK; 4 School of Health Sciences, University of Southampton, Southampton, UK; 5 Oxford Institute Nursing, Midwifery and Allied Health Research, Oxford Brookes University, Oxford, UK; 6 Bristol Urological Institute, North Bristol NHS Trust, Southmead Hospital, Bristol, UK; 7 Tyntesfield Medical Group, Brockway Medical Centre, Nailsea, Bristol, UK; 8 NIHR Bristol Biomedical Research Centre, University Hospitals Bristol and Weston NHS Foundation Trust and the University of Bristol, University Hospitals Bristol Education Centre, Bristol, UK; 9 Patient representative, UK; 10 Centre of Academic Primary Care, Population Health Sciences, Bristol Medical School, University of Bristol, Bristol, UK; 11 Population Health Sciences, Bristol Medical School, University of Bristol, Bristol, UK

## Abstract

**Objective:**

To determine whether a standardised and manualised care intervention in men in primary care could achieve superior improvement of lower urinary tract symptoms (LUTS) compared with usual care.

**Design:**

Cluster randomised controlled trial.

**Setting:**

30 National Health Service general practice sites in England.

**Participants:**

Sites were randomised 1:1 to the intervention and control arms. 1077 men (≥18 years) with bothersome LUTS recruited between June 2018 and August 2019: 524 were assigned to the intervention arm (n=17 sites) and 553 were assigned to the usual care arm (n=13 sites).

**Intervention:**

Standardised information booklet developed with patient and expert input, providing guidance on conservative and lifestyle interventions for LUTS in men. After assessment of urinary symptoms (manualised element), general practice nurses and healthcare assistants or research nurses directed participants to relevant sections of the manual and provided contact over 12 weeks to assist with adherence.

**Main outcome measures:**

The primary outcome was patient reported International Prostate Symptom Score (IPSS) measured 12 months after participants had consented to take part in the study. The target reduction of 2.0 points on which the study was powered reflects the minimal clinically important difference where baseline IPSS is <20. Secondary outcomes were patient reported quality of life, urinary symptoms and perception of LUTS, hospital referrals, and adverse events. The primary intention-to-treat analysis included 887 participants (82% of those recruited) and used a mixed effects multilevel linear regression model adjusted for site level variables used in the randomisation and baseline scores.

**Results:**

Participants in the intervention arm had a lower mean IPSS at 12 months (adjusted mean difference −1.81 points, 95% confidence interval −2.66 to −0.95) indicating less severe urinary symptoms than those in the usual care arm. LUTS specific quality of life, incontinence, and perception of LUTS also improved more in the intervention arm than usual care arm at 12 months. The proportion of urology referrals (intervention 7.3%, usual care 7.9%) and adverse events (intervention seven events, usual care eight events) were comparable between the arms.

**Conclusions:**

A standardised and manualised intervention in primary care showed a sustained reduction in LUTS in men at 12 months. The mean difference of −1.81 points (95% confidence interval −0.95 to −2.66) on the IPSS was less than the predefined target reduction of 2.0 points.

**Trial registration:**

ISRCTN Registry ISRCTN11669964.

## Introduction

Lower urinary tract symptoms (LUTS) relate to the storage and voiding of urine (box 1). The severity and prevalence of LUTS in men increases with age (as much as 30% in men older than 65 years),^1^ with greater numbers likely to be affected as the population ages. LUTS can have a substantial impact on quality of life,^2^ with problematic LUTS referred to as bothersome. Men usually present with a range of LUTS that can relate to storage, voiding, or post-voiding urinary symptoms, and most men are initially assessed and managed by their general practitioner. LUTS in men can be caused by obstruction of the prostate or bladder dysfunction, or both, but symptoms can also be influenced by lifestyle factors. The UK National Institute for Health and Care Excellence and the European Association of Urology recommend assessments to exclude serious medical conditions and to categorise and assess the impact of precise symptoms.^1^ ^3^ Assessment of LUTS is time consuming, however, and the level undertaken in general practice varies.^4^

Box 1 **Lower urinary tract symptoms in men**

Lower urinary tract symptoms (LUTS) in men can be caused by structural or functional abnormalities of the bladder, prostate gland, or urethra
Voiding LUTS are problems passing urine, such as hesitancy, slow urinary stream, and dribbling
Storage LUTS include urgency, increased urinary frequency, and nocturia. Storage LUTS can be due to increased urine volumes from high fluid intake or systemic conditions (cardiovascular, respiratory, renal, or endocrine)
Patients may experience one or more LUTS, and the severity of each symptom may not correlate with how much patients consider the symptoms to be bothersome
LUTS can be measured using the International Prostate Symptom Score (IPSS), with scores of 0-7 categorised as mild overall severity, 8-19 as moderate, and 20-35 as severe

NICE and the European Association of Urology recommend using conservative treatments for LUTS initially, including bladder training, advice on fluid intake, and lifestyle advice, although evidence on effectiveness of these measures is lacking.^1^ ^5^An NHS Evidence Update in 2012 indicated a role for self-management to treat LUTS, based on a post hoc analysis of a single centre randomised controlled trial of 140 men.^6^ ^7^ ^8^ NICE Clinical Guideline 97, however, recommends that a multicentre randomised controlled trial would be needed to determine effectiveness in clinical practice.^1^ Delivery of conservative treatments in primary care is also limited,^4^ which can result in men simply receiving drugs to treat prostate conditions, potentially being referred inappropriately to secondary care, or enduring persistent bothersome symptoms.

As provision for LUTS in men in primary care is inconsistent, primary care health professionals require practical resources to support the assessment of urinary symptoms and to enhance patient engagement with conservative management interventions. We aimed to address this need in the Treating Urinary Symptoms in Men in Primary Healthcare (TRIUMPH) study. The main objective was to determine whether a standardised and manualised care intervention in men in primary care in the UK could achieve superior improvement of LUTS compared with usual care. The primary outcome was overall International Prostate Symptom Score (IPSS) measured 12 months after participants had consented to participate in the study.

## Methods

The TRIUMPH study was a multicentre, pragmatic, two arm cluster randomised controlled trial in UK primary care. The trial was conducted in 30 general practice sites, with participants recruited from June 2018 to August 2019. The trial design included an internal pilot recruitment phase of four months’ duration, primarily to verify that recruitment was achievable before progression to the main phase of the trial. Specification of an exact figure for the number of eligible participants required by general practices to take part in the trial, as determined by a pre-randomisation practice database search, was removed for the main phase of the trial to allow flexibility according to patients’ response rates.

The trial protocol was submitted for publication before recruitment ended, and the trial was registered prospectively on 12 April 2018.^9^ The statistical analysis plan was finalised in July 2020, before completion of follow-up (August 2020).^10^

### General practice sites

Clinical Research Networks recruited the general practices from across the National Institute for Health Research (NIHR, now referred to as the National Institute for Health and Care Research) West of England and Wessex Clinical Research Network regions. Practices were eligible if they had an adequate number of eligible patients determined by a pre-randomisation practice database search (to achieve a target recruitment of 35 participants at each site), with suitable space for treatment rooms and availability for training of healthcare professionals and baseline visits. In the final selection of practices for randomisation, we also considered representative practice list size, social deprivation score (index of multiple deprivation determined using the general practice’s postcode), and preference for how the intervention would be delivered (practice staff or trial research nurses) if the practice was randomised to the intervention arm.^9^ Groups of practices with shared nurse resources were randomised as a single site.

### Participants

Men aged 18 years or older who had presented to primary care with LUTS within the past five years according to general practice records and were currently experiencing at least one bothersome LUTS were potentially eligible for the study. Men were excluded if they lacked capacity to consent, were unable to pass urine without a catheter (indwelling or intermittent catheterisation), had a relevant neurological disease or referral, were undergoing urological testing for LUTS, were being treated for prostate or bladder cancer, had undergone prostate surgery, had poorly controlled diabetes mellitus, were recently referred or currently under urology review, had visible haematuria, or were unable to complete trial assessments in English.

The general practices conducted a single database search designed specifically for the trial to identify potentially eligible patients, and the general practitioners manually verified the findings using electronic medical records. To avoid any bias in patient selection, each site conducted a single mail out to potentially eligible patients before notification of its randomisation status. While masked to the allocation of the practice and to avoid bias, NIHR Clinical Research Network nurses or clinical practitioners trained by the trial team telephoned patients expressing an interest in taking part in the study. Calls were conducted to confirm eligibility, particularly the subjective criterion of whether LUTS were currently bothersome to the patient, as initial screening by the general practices only identified men coded with LUTS within the preceding five years. The telephone calls also ensured that patients understood the study, answered any questions, and confirmed their willingness to participate in the study.

Patients deemed willing and eligible completed a postal consent form and questionnaire containing baseline measures. All patients received the same consent form and questionnaires, but those in the intervention arm also received a bladder diary to be completed before their face-to-face visit for assessment of symptoms. Participants remained blinded to their treatment arm while completing baseline measures and were not aware that completion of the bladder diary indicated randomisation to the intervention arm. Participants assigned to receive the intervention did not have sight of the booklet until after consent had been obtained, and participants assigned to receive usual care remained unaware of the content of the booklet throughout the trial.

### Intervention

The TRIUMPH intervention employed a standardised information booklet. Participants were directed to information within the booklet that was applicable to them via healthcare professional assessment and discussion, providing the manualised element of the intervention. In collaboration with patients, healthcare professionals, and health psychologists, the booklet was developed for the study from patient information sheets produced by the British Association of Urological Surgeons (see supplementary file). The booklet provides targeted guidance on conservative and lifestyle interventions for LUTS in men and is water resistant and able to lie flat for ease of use when opened. Sections are tabbed and colour coded for specific LUTS symptoms and advice.

Depending on site preference, participants received the booklet from a general practice clinical nurse, research nurse, healthcare assistant, or trial research nurse. The trial research nurses provided training and ongoing support to the healthcare professionals delivering the intervention. Before the participants attended for an intervention visit, the healthcare professional reviewed the participants’ baseline urinary symptoms, utilising their completed IPSS, International Consultation on Incontinence Questionnaire Urinary Incontinence-Short Form, and International Consultation on Incontinence Questionnaire bladder diary. During this visit the healthcare professional discussed the participants’ individual symptoms and how bothersome they were to the participant. The healthcare professionals were provided with decision tools (see supplementary file) enabling them to direct participants to relevant sections of the booklet on the basis of reported symptoms. A maximum of three sections were recommended to each participant and tabbed with discreet stickers. The sections provided advice on drinks and liquid intake, controlling an urgent need to urinate, exercises for the pelvic floor muscles to help stop bladder leakage, emptying the bladder as completely as possible, getting rid of the last drops of urine, and reducing sleep disturbance caused by the need to urinate at nighttime.

To encourage and gauge adherence to the intervention, healthcare professionals followed-up the participants by telephone at one week and then by telephone, email, or text at four and 12 weeks, according to the participants’ preference. Participants retained the intervention booklet thereafter. Participants in the intervention arm continued to receive usual care for LUTS from their doctor.

The usual care practices were requested to continue standard local management for LUTS. At the end of the study, participants in the usual care arm were provided with the booklet along with a summary of the trial’s results.

Participants in both randomised groups were provided with progress updates of the study at three and nine months via a newsletter to maintain engagement with the trial and encourage completion of follow-up questionnaires.

### Outcomes

The primary outcome measure was the validated patient reported IPSS at 12 months after consent to participate in the study—a score that is extensively used in LUTS research and widely employed in urology services.^11^ IPSS scores range from 0 to 35, with higher scores indicating more severe symptoms. The endpoint of 12 months was chosen to measure whether the effect of the TRIUMPH intervention on LUTS was sustained after the initial 12 week delivery period.

Secondary outcomes collected by questionnaire at baseline and six and 12 months after consent comprised the IPSS quality of life (LUTS related quality of life score, six and 12 months), the IPSS (overall score for urinary symptoms, six months), the International Consultation on Incontinence Questionnaire Urinary Incontinence-Short Form symptoms score (six and 12 months,^12^ which supplements the IPSS with measurement of incontinence and post-void dribble), the EQ-5D (five level version of the EuroQoL index, EQ-5D-5L, measure of health status, six and 12 months, used to create quality adjusted life years for the health economic evaluation, which will be reported separately),^13^ and the Brief Illness Perception Questionnaire (measuring participants’ cognitive and emotional perception of their LUTS, and which was modified slightly, with developers’ permission, to ask about “urinary symptoms” rather than “illness,” six and 12 months).^14^

The number of LUTS related adverse events (prespecified as urinary tract infections, catheterisations, urinary retention, prostatitis), deaths, and the number of referrals to secondary care (urology) at 12 months post-consent were extracted from primary care electronic medical records through trial specific automated database searches. These searches were conducted a minimum of one month after the final participant for each site had completed follow-up. The sites provided the central trial team with anonymised data extracts for analysis.

Case report forms were specifically designed for this study. Healthcare professionals completed the forms for the intervention arm only—at the intervention visit and during the 12 week treatment phase to collect details on sections of the booklet recommended to participants, and feedback on the booklet.

The health economic analysis of this trial will be reported elsewhere.

### Sample size calculation

TRIUMPH was designed to detect a mean difference of 2.0 points between arms on the IPSS at 12 months post-randomisation with 90% power, as this is the mean decrease in IPSS among men who rate their condition as slightly improved when the baseline scores are <20 points.^15^ As outlined in the study protocol,^9^ this value is less than the previously observed minimal clinically important difference of 3 points for IPSS^16^ but allows for a difference in just one symptom. Based on a scoping search of local general practices, we estimated we would need a mean cluster size of 35 participants and proposed an estimated intraclass correlation coefficient of 0.05 based on other studies in primary care.^17^ The experience from previous trials suggested it would be prudent to allow for up to 30% loss to follow-up. On this basis, we estimated that 840 participants would be needed from at least 24 sites to achieve 90% power.

Early in the study, however, we observed variability between sites in the number of participants recruited, thus necessitating a revision of the sample size calculation. Using recruitment data available at the time, we estimated that the mean number of participants who would need to provide consent at each site would be 26 and that the coefficient of variation of the mean cluster size would be 0.26. Ignoring clustering and loss to follow-up, we determined that 263 participants in total would be required to detect a difference of 2 units in IPSS with 90% power assuming a common standard deviation of 5. Our updated design effect assumed an intraclass correlation coefficient of 0.05; a mean of 26 patients consenting in each site but only 70% providing primary outcome data resulting in a mean cluster size of 18.2; and a 0.26 coefficient of variation in cluster sizes. Under these revised assumptions, the design effect is 1.92, meaning that 506 participants in total would be required to provide primary outcome data. Given our assumed loss to follow-up, 724 patients needed to provide consent to participate in the study, and as each site was expected to obtain consent for 26 patients, this translated to 28 sites in total. Allowing for some practices not to perform as expected, 30 were ultimately recruited in agreement with the trial management group, steering committee, and funder.

### Randomisation and blinding

General practice sites were the units of allocation. A statistician blinded to the identity of practices randomised them on a 1:1 basis to deliver either the TRIUMPH intervention or usual care. Randomisation was performed after the practices had completed screening of patients and sent out invitations to eligible participants. Randomisation was minimised by centre (West of England and Wessex Clinical Research Network regions), practice size (number of registered patients), and area level deprivation of the practice (index of multiple deprivation score). We incorporated a random element into the minimisation procedure such that there was a 40% probability that allocation was random, with a 50-50 chance of practices being allocated to either arm. Area level deprivation assessed at the lower super output area level (geography comprising between 400 and 1200 households) can estimate deprivation for individuals (using home postcodes to identify the lower super output area level), but middle layer super output area level (geography made up of four or five lower super output area level) data better reflect the area level deprivation of general practices because the catchment area of a general practice is generally wider than the area covered by the lower super output area level.^18^ As such, we mapped the postcodes of the general practices onto lower super output area level then middle layer super output area level. Population averaged index of multiple deprivation scores (2015) were then calculated based on the scores of lower super output area level within each middle layer super output area level.

To minimise selection and recruitment bias, staff who conducted telephone calls for patient eligibility were blinded to practice allocation. Participants were blinded to their allocation until they had completed the baseline questionnaire and provided a signed consent form.

### Safety

General practices were responsible for reporting serious adverse events experienced by the participants; and participants were also asked to report any inpatient stays in their follow-up questionnaires, which would prompt review by their general practitioner. The study independent data monitoring committee reviewed reported serious adverse events every six months. All other adverse events were collected from participants’ primary care electronic medical records, as part of the secondary outcomes.

### Statistical analysis

Statistical analyses were conducted with all consenting participants retained in the randomised arm of their general practice. Baseline characteristics at individual and practice level were summarised using means, standard deviations, medians (interquartile ranges), or number (percentage) depending on the nature and distribution of the data.

The primary analysis of IPSS at 12 months was conducted on a modified intention-to-treat (ITT) basis, and comparisons between treatment arms were made using mixed effect multilevel linear models (individuals (level 1) nested within general practices (level 2)) adjusting for individual level baseline IPSS and practice level variables used in the randomisation based on those providing non-missing data for the variables included in the model. The results are presented as the mean difference between arms, 95% confidence interval, P value, and model intraclass correlation (95% confidence interval).

The secondary outcomes were also analysed on a modified ITT basis. IPSS at six months were analysed using a mixed effect multilevel linear model (individuals (level 1) nested within general practices (level 2)) adjusting for individual level baseline IPSS and practice level variables used in the randomisation. Additionally, and separately, a repeated measures analysis was conducted using a repeated measures linear mixed model (IPSS at six and 12 months (level 1), nested within participants (level 2) and nested within general practices (level 3)) adjusting for individual level baseline IPSS scores and practice level variables used in the randomisation. Minimal clinically important differences for LUTS in men are not established in the literature for the secondary outcomes included.

We planned seven sensitivity analyses to assess the robustness of the primary analysis to varying assumptions. Each of these sensitivity analyses was compared with the primary analysis.

• Descriptive statistics were used to assess whether baseline characteristics were balanced between the two arms, and, if differences were observed, the primary analysis would be re-run adjusting for those imbalanced variables. This analysis was not performed, however, because we found no evidence of imbalance in variables not already included in the primary analysis.

• To allow for possible clustering of outcomes within nurses and healthcare assistants delivering the intervention, we grouped patient level data according to the combination of practice and nurse or healthcare assistant delivering the intervention. The primary analysis was then re-run using a single random effect for this level of clustering.

• Although the target recruitment was reached before the covid-19 outbreak, we wanted to allow for any influence of the outbreak and subsequent lockdowns on participation and symptom reporting. To do this we repeated the primary analysis including a binary variable for whether or not the outcome measure was taken before or after 11 March 2020 (the date the World Health Organization declared the outbreak a pandemic).

• A small number of recruited participants were subsequently found to be ineligible. They were included in the primary analysis, and a sensitivity analysis was performed excluding those individuals.

• A series of per protocol analyses were performed using different definitions of protocol compliance (see supplementary table S1).

• Recognising the biases inherent in per protocol analyses we also performed a complier average causal analysis. Compliers were those participants who received the intervention booklet by the time of follow-up for the primary outcome. The estimates for complier average causal analysis were obtained using instrumental variable regression with the same variables used in the primary analysis, with the randomised arm as the instrumental variable and an indicator variable for compliance.

• We explored the impact of missing primary outcome data using different assumptions for missingness: “best” and “worst” case scenarios as well as multiple imputation by chained equations to impute missing data.

To explore whether the effectiveness of the intervention on the primary outcome differed by participant subgroup, we performed four prespecified subgroup analyses. In each case, effect modification was assessed by including a subgroup-treatment interaction term and performing a likelihood ratio test comparing the model with and without the interaction term. A significance level of 5% was used, but as these analyses were not statistically powered, they should be interpreted with caution. Subgroup analyses assessed whether effectiveness differed by the nature of LUTS at baseline measured by the ratio of the IPSS voiding score to the storage score; whether a practice nurse, healthcare assistant, or trial nurse delivered the intervention; the participant’s preferred method of contact at baseline; and the number of contacts between the nurse or healthcare assistant and participant at intervention practices.

### Patient and public involvement

Patient and public involvement (PPI) representatives have been involved at all stages from a patient co-applicant at the grant application stage to help shape the project to patient representative members of our trial management group and trial steering committee who helped steer the trial. Wider patient advisory group meetings were also held over the course of the study. Development of the TRIUMPH intervention booklet was one of the key roles for PPI, resulting in important changes to aid clarity and usability and recommendations on what patients would consider a manageable level of advice to follow. PPI review of our patient-facing study materials was also undertaken, including patient questionnaires to assess clarity and participant burden, newsletters, and the study website. Further PPI involvement has included discussion of some of our initial qualitative findings related to men’s experiences of the patient pathways for LUTS within the NHS, as well as routes for implementation and dissemination, and patients will continue to be involved.

## Results

**Fig 1:**
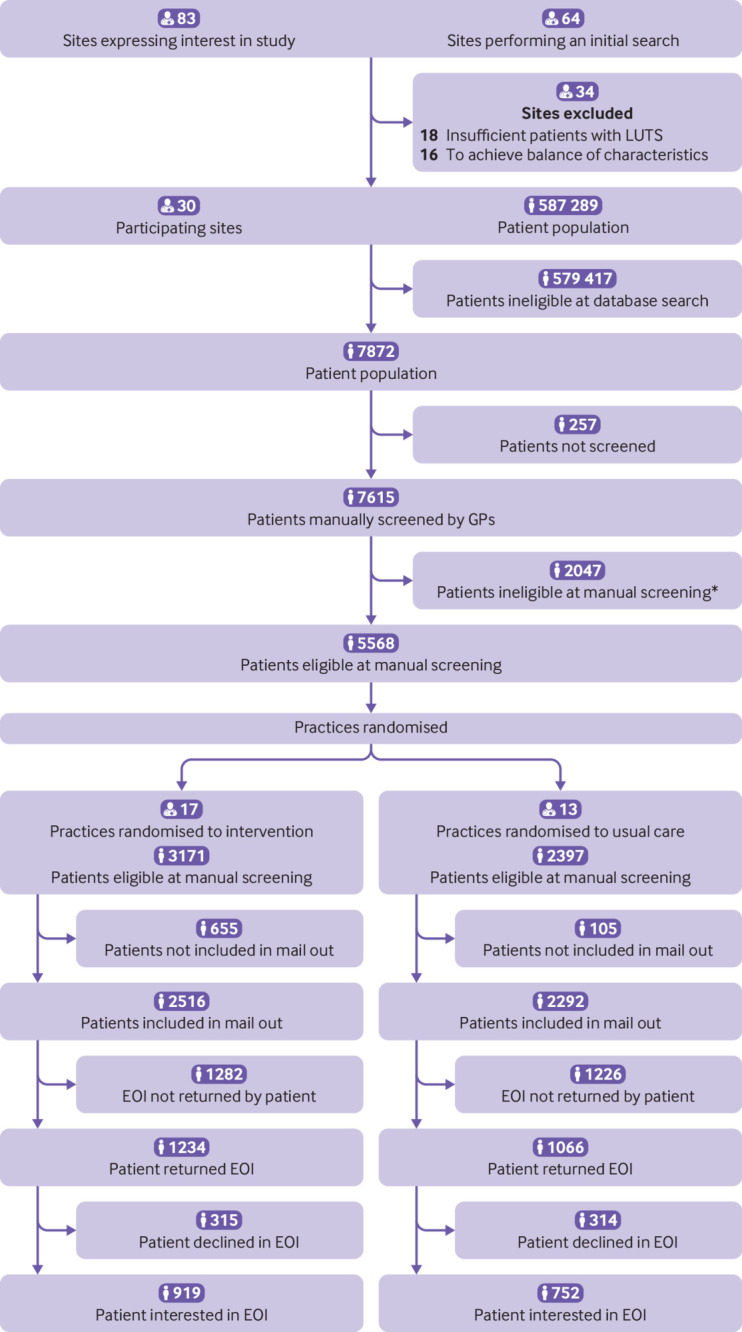
Flow of practice sites and participants through study. *See supplementary table S1 for reasons for exclusion. EOI=expression of interest; GP=general practitioner; LUTS=lower urinary tract symptoms

**Fig 2:**
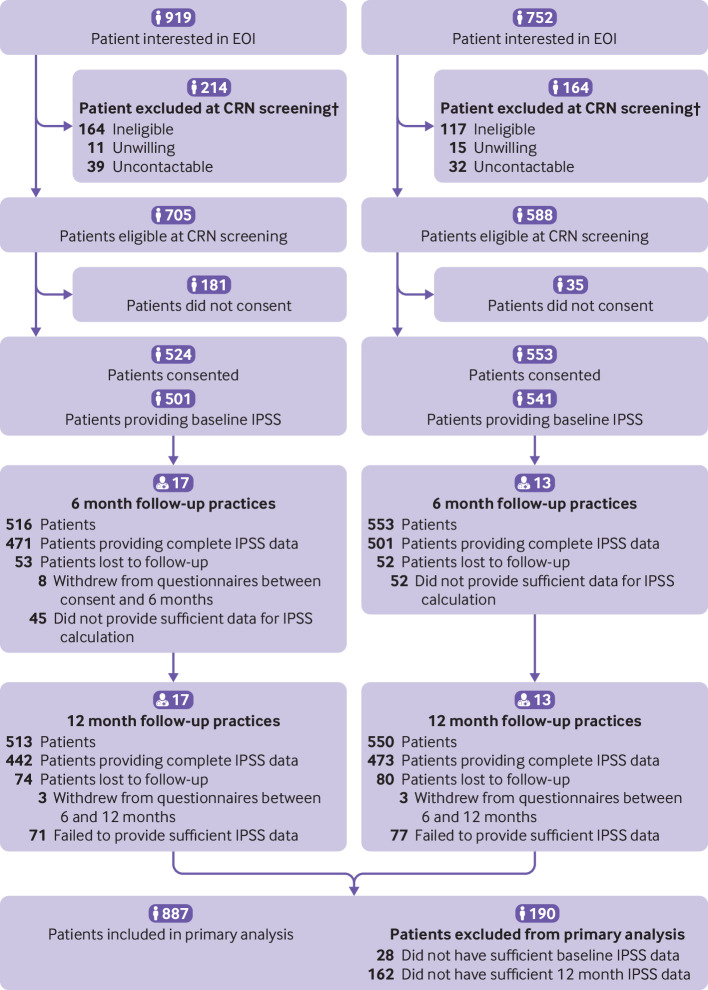
Flow of practice sites and participants through study as continuation of figure 1. †Participants remained blinded to treatment arm through all screening processes, until point of consent. CRN=clinical research network; EOI=expression of interest; IPSS=International Prostate Symptom Score

Thirty primary care sites (32 general practices; one group of three practices were randomised as a single site) were recruited and all contributed to the intention-to-treat analysis (fig 1 and fig 2); 17 were randomised to the intervention and 13 to usual care. At the time of recruitment, the general practices provided estimated (pre-screening) practice list sizes ranging from 7600 to 48 623 patients (mean=19 576) reflecting some of the larger practices in the Wessex and West of England regions (combined regional median practice size in June 2019: 9440). Area level index of multiple deprivation scores ranged from 4.22 to 33.62 for practices, and the mean was slightly higher in usual care practices, suggesting greater levels of socioeconomic deprivation than in intervention practices (table 1).

**Table 1:** Characteristics of practice sites and participants at baseline by intervention group. Values are number (percentage) unless stated otherwise

	Intervention arm			Usual care arm	
	No*	Estimate		No*	Estimate
**Site level characteristics**					
Total No of sites	17	—		13	—
Mean (SD) practice size	17	20 694 (9714)		13	18 114 (7998)
Mean (SD) No of participants providing consent per site	17	31 (12.05)		13	43 (12.71)
Mean (SD) area level deprivation of practice based on postcode	17	11 (5.02)		13	16 (8.39)
**Participant level characteristics**					
Total No of participants	524	—		553	—
**Personal characteristics**					
Mean (SD) age (years); (min-max)	524	68.9 (9.3) (32-94)		553	68.4 (9.2) (30-95)
Ethnicity:					
White	522	513 (98.3)		550	542 (98.6)
Black, Asian, mixed, or other		8 (1.5)			5 (0.9)
Disclosure declined		1 (0.2)			3 (0.6)
Marital status:					
Single	517	21 (4.1)		543	25 (4.6)
Married or civil partnered		436 (84.3)			455 (83.8)
Divorced		31 (6.0)			32 (5.9)
Widowed		27 (5.2)			28 (5.2)
Disclosure declined		2 (0.4)			3 (0.6)
Index of multiple deprivation fifth:					
1st (most deprived)	506	17 (3.4)		525	21 (4.0)
2nd		33 (6.5)			37 (7.0)
3rd		67 (13.2)			106 (20.2)
4th		141 (27.9)			136 (25.9)
5th (least deprived)		248 (49.0)			225 (42.9)
Median (IQR) index of multiple deprivation score		8.80 (5.75-13.71)			9.89 (6.21-15.45)
**Clinical characteristics**					
Mean (SD) height (cm) (min-max)	518	176.72 (6.77) (152.40-198.12)		550	176.93 (7.41) (157.48-208.28)
Mean (SD) weight (kg) (min-max)	510	83.36 (14.45) (55.02-152.41)		549	83.89 (14.29) (53.98-136.98)
Mean (SD) body mass index (min-max)	508	26.71 (4.40) (18.91-52.31)		549	26.76 (4.00) (17.57-42.18)
No of comorbidities:					
0	478	151 (31.6)		544	171 (31.4)
1		160 (33.5)			197 (36.2)
>1		167 (34.9)			176 (32.3)
Test results in 6 months pre-baseline:					
Urine analysis: abnormal	79	1 (1.3)		52	2 (3.9)
Kidney function: eGFR (mL/min/1.73m^2^)	170	—		215	—
eGFR measures:					
Mean (SD) eGFR	170	73.5 (15.7)		215	74.6 (13.2)
Median (IQR) eGFR		76.5 (65-87)			75 (66-87)
Min-max eGFR		28-98			36-100
CKD stages based on recent eGFR (mL/min/1.73m^2^):					
≥90: normal	170	28 (16.5)		215	33 (15.4)
90-60: stages G1-G2		114 (67.1)			154 (71.6)
30-59: stage G3		27 (15.9)			28 (13.0)
<30: stages G4-G5		1 (0.6)			0 (0)
No of GP consultations in 12 months before baseline:					
Mean (SD)	478	4.4 (3.7)		544	4.8 (5.0)
Median (IQR)		4 (2-6)			4 (2-6)
Min-max		0-23			0-58
Referrals to urology in 12 months pre-baseline:					
0	478	464 (97.1)		544	525 (96.5)
1		14 (2.9)			19 (3.5)
>1		0			0
**Patient reported symptoms and quality of life**					
Mean (SD) IPSS (min-max):					
Incomplete emptying	512	1.7 (1.5) (0-5)		549	1.9 (1.5) (0-5)
Frequency	514	2.7 (1.3) (0-5)		551	2.9 (1.4) (0-5)
Intermittency	514	1.9 (1.6) (0-5)		549	2.0 (1.7) (0-5)
Urgency	513	2.1 (1.6) (0-5)		549	2.3 (1.7) (0-5)
Weak stream	510	1.9 (1.5) (0-5)		549	2.0 (1.7) (0-5)
Straining	513	0.8 (1.2) (0-5)		548	1.0 (1.3) (0-5)
Nocturia	516	2.6 (1.4) (0-5)		551	2.4 (1.2) (0-5)
Mean (SD) total IPSS (min-max)	501	13.6 (5.8) (1-33)		541	14.6 (6.6) (2-34)
Symptom severity by IPSS:					
≤7: mild	501	76 (15.2)		541	74 (13.7)
8-19; moderate		342 (68.3)			338 (62.5)
≥20: severe		83 (16.6)			129 (23.8)
Mean (SD) IPSS quality of life score† (min-max)	516	3.5 (1.2) (0-6)		551	3.6 (1.1) (0-6)
Mean (SD) ICIQ-UI-SF total score (min-max)	513	3.6 (3.6) (0-14)		542	3.9 (3.7) (0-15)
ICIQ-UI-SF urine leakage‡:					
Never	523	185 (35.4)		553	161 (29.2)
Before getting to the toilet		205 (39.2)			237 (42.9)
When coughing/sneezing		24 (4.6)			24 (4.4)
When asleep		12 (2.3)			15 (2.7)
During physical activity		23 (4.4)			27 (4.9)
After urinating/when dressed		175 (33.5)			205 (37.1)
No obvious reason		36 (6.9)			42 (7.6)
All the time		1 (0.2)			1 (0.2)
Mean (SD) B-IPQ total score (min-max)	440	38.7 (11.0) (1-75)		478	39.4 (10.4) (6-72)
Bladder diary§:					
Incontinence	502	100 (19.9)		—	—
Urgency	507	364 (71.8)		—	—
Nocturia¶	261	222 (85.1)		—	—

B-IPQ=Brief Illness Perception Questionnaire; CKD=chronic kidney disease; eGFR=estimated glomerular filtration rate; IQR=interquartile range; ICIQ-UI-SF=International Consultation on Incontinence Questionnaire Urinary Incontinence-Short Form; IPSS=International Prostate Symptom Score; SD=standard deviation.

* Number providing non-missing data at baseline.

† Question asked: “If you were to spend the rest of your life with your urinary condition the way it is now, how would you feel about that?”

‡ Question asked: “When does urine leak?”

§ Bladder diary completed as part of initial assessment in intervention arm only.

¶ For description purposes at baseline, nocturia is defined as waking to urinate at least once on two nights or to urinate twice or more. When data on waking or sleeping were not provided by participants, the variable was set to missing.

The general practices identified 7872 potentially eligible patients from database searches. A random selection of 160 patients were not screened owing to agreed limits on screening numbers by large practices, and 97 patients were not screened owing to practice capacity. Of the remaining 7615 patients manually screened by their doctors, 2047 were ineligible (fig 1) (see supplementary table S2). Of the eligible patients identified, 4808 were invited to join the study, with a maximum of 150 (pilot phase) or 220 (main phase) invited patients per site, to avoid over-representation of larger sites.

Of the 4808 participants invited, 2300 (47.8%) responded to the single invitation (no reminders were sent out). Of those who responded, 1671 were interested in taking part (72.6%). On further screening for eligibility (in particular for current bothersome LUTS), 1293 (77.4%) of those interested were eligible. Of these, 524 participants were recruited from intervention sites and 553 from usual care sites (83% of those interested and eligible) (fig 2). Patients remained blinded to their assignment arm until after consent had been obtained. On average, men were in their late 60s, with a predominance of white and married or civil partnered men (table 1). The distribution of clinical characteristics was comparable between treatment arms. The median number of doctor consultations in the 12 months before baseline was the same in both arms, but the proportion with a referral to urological services in that period was slightly lower in the intervention arm (intervention 2.9%, usual care 3.5%). Baseline IPSS was slightly lower in the intervention arm than usual care arm, indicating a lower symptom burden; but this was not reflected in the International Consultation on Incontinence Questionnaire Urinary Incontinence-Short Form focused on incontinence. Quality of life (IPSS quality of life) was comparable between the two arms, as was patients’ perception of their LUTS (measured using the Brief Illness Perception Questionnaire).

### Primary outcome: IPSS at 12 months

Overall, 915 participants (85.0% of those randomised) provided primary outcome data, of whom 887 provided sufficient baseline data to be included in the analysis. Some improvement in LUTS occurred at 12 months in both arms, but this was greater in the intervention arm (difference in IPSS −1.81 (95% confidence interval −2.66 to −0.95), P<0.001) after adjustment for baseline values and minimisation variables (table 2).

**Table 2:** Analyses of treatment effectiveness on patient reported primary outcome and secondary outcomes

	Intervention			Usual care			Adjusted analysis*		
	No†	Mean (SD) (min-max)		No†	Mean (SD) (min-max)		Difference (95% CI); P value	No analysed	ICC (95% CI)
**Primary outcome**									
IPSS: 12 months‡	442	11.6 (6.2) (1-35)		473	13.9 (6.8) (0-32)		−1.81 (−2.66 to −0.95); <0.001	887	0.011 (0.001 to 0.086)
**Secondary outcomes**									
IPSS (range 0-35):									
6 months‡	471	11.5 (6.1) (1-35)		501	13.8 (6.6) (1-32)		−1.68 (−2.34 to −1.02); <0.001	942	<0.001 (<0.001 to <0.001)
6 and 12 months (repeated measures analysis)§	913	11.6 (6.2) (1-35)		974	13.8 (6.7) (0-32)		−1.70 (−2.35 to −1.05); <0.001	1829	0.005 (0.0002 to 0.115)
ICIQ-UI-SF score (range 0-21):									
6 months‡	476	3.6 (3.5) (0-15)		504	4.5 (4.1) (0-20)		−0.53 (−1.02 to −0.04); 0.04	961	0.022 (0.007 to 0.072)
12 months‡	453	3.7 (3.6) (0-18)		480	4.5 (4.1) (0-18)		−0.74 (−1.15 to −0.33); <0.001	915	<0.001 (<0.001 to <0.001)
IPSS quality of life score (range 0-6):									
6 months‡	483	3.0 (1.2) (0-6)		511	3.35 (1.25) (0-6)		−0.29 (−0.43 to −0.15); <0.001	984	NE
12 months‡	463	2.9 (1.3) (0-6)		483	3.3 (1.25) (0-6)		−0.34 (−0.50 to −0.18); <0.001	937	0.004 (<0.001 to 0.274)
B-IPQ score (range 0-90):									
6 months‡	450	33.4 (11.9) (2-73)		430	38.3 (11.5) (1-76)		−5.34 (−6.69 to −3.99); <0.001	777	<0.001 (<0.001 to <0.001)
12 months‡	419	33.8 (12.0) (0-69)		427	38.4 (12.2) (0-71)		−4.78 (−6.31 to −3.25); <0.001	746	NE

B-IPQ=Brief Illness Perception Questionnaire; ICC=intraclass correlation; CI=confidence interval; ICIQ-UI-SF=International Consultation on Incontinence Questionnaire Urinary Incontinence-Short Form; IPSS=International Prostate Symptom Score; NE=not estimable; SD=standard deviation.

Higher scores for IPSS, ICIQ-UI-SF, IPSS quality of life, and B-IPQ reflect more severe symptoms, greater impact on quality of life, or participants’ poorer perception of their LUTS.

* Adjusted for baseline scores and minimisation variables.

† Number of participants in each arm providing non-missing outcome data except in repeated measures analysis of IPSS (six and 12 months) where this reflects the number of repeated measures of IPSS in each arm.

‡ Analysed using mixed effect multilevel linear model (individuals (level 1) nested within general practices (level 2)) adjusting for individual level baseline outcome measure and practice level variables used in the randomisation.

§ Analysed using a repeated measures linear mixed model (IPSS at six and 12 months (level 1), nested within participants (level 2) and nested within general practices (level 3)) adjusting for individual level baseline IPSS and practice level variables used in the randomisation.

A planned sensitivity analysis accounting for clustering by nurse or healthcare assistant had little effect on the primary outcome results (difference −1.79 (95% confidence interval −2.53 to −1.06), P=0.004), nor did excluding three participants who were found to be ineligible after follow-up began (difference −1.81 (−2.65 to −0.96), P<0.001) or adjusting for whether the outcome data were collected during the covid-19 pandemic (difference −1.89 (−2.69 to −1.09), P<0.001). Imputation of missing data also yielded comparable results to the complete case analysis (table 3).

**Table 3:** Sensitivity analysis comparing results of ITT analysis of complete cases with ITT analyses, with missing IPSS data imputed

	No imputed		No used in analysis	Overall mean (SD)	Difference in means* (95% CI)	P value
	Intervention arm	Usual care arm				
Complete case ITT	0	0	887	12.79 (6.64)	−1.81 (−2.66 to −0.95)	<0.001
Best case scenario	77	78	1042	10.89 (7.63)	−1.89 (−2.89 to −0.88)	<0.001
Worst case scenario	77	78	1042	14.82 (7.82)	−1.14 (−2.08 to −0.20)	0.02
MICE†	82	80	1077	—	−1.61 (−2.57 to −0.66)	0.001

CI=confidence interval; IPSS=International Prostate Symptom Score; SD=standard deviation ITT=intention to treat; MICE=multiple imputation by chained equations; SD=standard deviation.

* Analysis adjusted for baseline IPSS and minimisation variables.

† Data are imputed using IPSS at baseline and six months, treatment arm, practice size, centre, and deprivation (index of multiple deprivation). To allow for clustering in Stata, imputations were performed separately for each practice.

### Secondary outcomes

The difference in mean IPSS was also evident between the two arms at six months, although slightly less than at 12 months, and in the repeated measures analysis of IPSS (six and 12 months) (table 2). Incontinence scores were also lower in the intervention arm compared with usual care arm at six and 12 months (as assessed with the International Consultation on Incontinence Questionnaire Urinary Incontinence-Short Form), with the improvement in the intervention arm being greater at 12 months than at six months (table 2). Mean IPSS LUTS specific quality of life scores at six and 12 months were near the middle of the range of scores for this measure but showed evidence of small differences between the arms (table 2). Patient perception of their LUTS (measured using the Brief Illness Perception Questionnaire) showed a greater improvement in the intervention arm compared with usual care arm at both six and 12 months (table 2).

Similar proportions of men were referred to secondary care over the following 12 months (intervention 7.3% (35/478); usual care 7.9% (43/544)). After adjusting for randomisation variables and pre-baseline referrals, a difference between the arms was not evident (adjusted odds ratio 0.91 (95% confidence interval 0.51 to 1.62); P=0.76; intraclass correlation 0.02 (95% confidence interval 0.001 to 0.41)).

A similar, small number of LUTS related adverse events and deaths were reported in both arms (table 4). All serious adverse events that occurred during the study were unrelated to the intervention, such as those affecting a separate organ system (eg, bilateral epistaxis), with the exception of five that were deemed unlikely to be related to the intervention (see supplementary table S3).

**Table 4:** Expected adverse events identified from search of general practice electronic medical records

Adverse event	Intervention arm			Usual care arm	
	No of patients	No of events per patient		No of patients	No of events per patient
Prostatitis	2	1 and 4		2	1 and 6
LUTS related urinary tract infection	2	3 and 2		3	1 each
Urinary retention	1	1		2	2 and 4
Catheterisation	0	—		0	—
Death	2	—		1	—

LUTS=lower urinary tract symptoms.

### Subgroup analyses

No effect modification was found when including the ratio of storage to voiding LUTS at baseline as a continuous interaction term in the model of IPSS at 12 months (P=0.971). Similarly, distinguishing between those men who received the intervention via a study nurse (n=249) or a practice nurse or healthcare assistant (n=190) also showed no evidence of difference (P=0.387). Weak evidence of effect modification (P=0.094) was, however, found in relation to how intervention participants preferred follow-up by the clinical team (telephone=310; text or email=121), with those opting for contact by text or email showing a greater improvement compared with usual care than those opting for contact by telephone.

### Intervention delivery

Almost all the participants at intervention sites received the booklet (98.5%) and 91.7% received all three planned follow-up contacts, with most (79.0%) received in the protocolised format (week 1 by telephone, weeks 4 and 12 as preferred by participants). Given the high level of fidelity to the intervention, numbers deviating from protocolised follow-up were small and thus the planned per protocol analyses were underpowered, although findings were consistent with a greater change in IPSS at 12 months in the intervention arm (supplementary table S1). As only nine participants in the intervention arm were deemed to have been non-adherent, the planned complier average causal analysis was not performed.

## Discussion

This large, pragmatic randomised controlled trial in primary care showed that a range of bothersome LUTS improved over 12 months in men with mean IPSS scores regarded as reflecting moderate severity LUTS, using a standardised booklet and manualised approach to symptom management. The mean patient reported primary outcome (IPSS) was 1.81 points lower in the intervention arm than usual care arm. The secondary outcomes measured using the International Consultation on Incontinence Questionnaire and IPSS quality of life also showed improvement against usual care, demonstrating the overall impact on LUTS through incontinence, post-void dribble, and quality of life. In addition, participants’ perception of their LUTS improved over 12 months in the intervention arm (measured using the Brief Illness Perception Questionnaire). Referral rates to urology did not differ greatly between the arms. Adverse events also did not differ greatly between the arms.

The response rate of patients invited to participate in the study was 48%, which could be because those men who were historically coded as having LUTS in primary care in the previous five years were no longer experiencing bothersome symptoms, or that inaccuracies were present in the coding. In addition, only a single invitation was sent, with no reminder. Of those who responded, 73% were interested in taking part. Response rates were unrelated to acceptability of the intervention as men were blinded to their randomisation group until they had consented to the study, and without sight of the intervention booklet.

The mean IPSS at baseline was 13.6/14.6 in the two randomised groups, which is moderate by the accepted symptom severity categories (scores 8-19). The TRIUMPH study accepted men who were still experiencing any bothersome LUTS despite having consulted their doctor in the previous five years. This included storage or voiding LUTS, post-void dribble, and monosymptomatic nocturia (a symptom that can be caused by a wide range of medical conditions unrelated to the lower urinary tract).^19^ ^20^ Achieving improvement of symptoms in such a mixed population, using assessments and guidance in the form of a booklet provided by nurses or healthcare assistants, is challenging. The mean reduction in IPSS was 1.81 points greater than that obtained with usual care and was sustained for at least nine months beyond the final healthcare professional input into the intervention. Hence a considerable number of men saw improvement in symptoms at low risk and with low requirement for doctor’s input.

The target reduction of 2.0 points on which TRIUMPH was powered was less than the more generally used minimal clinically important difference of 3.0 points for the IPSS,^16^ as the threshold change for “slight improvement” in symptoms is affected by baseline IPSS.^15^ A minimal clinically important difference of 2.0 points is appropriate where baseline IPSS is <20. The study pragmatically included LUTS in all in its manifestations, potentially including men with just one symptom requiring treatment (eg, nocturia). For such men, the baseline IPSS could be as low as 2 (ie, nocturia twice nightly, the severity of nocturia generally accepted as impairing quality of life).^19^ These men could see improved quality of life when the severity of their nocturia was reduced to once nightly^21^ (ie, a reduction in IPSS from 2 to 1).

The observed reduction of 1.81 (95% confidence interval −2.66 to −0.95) was smaller than the predefined target reduction of 2.0 points, thus the improvement in symptoms related to the intervention may be small.^10^ The reduction in symptom score was relative to usual care, where a small overall reduction in IPSS was also seen at a year. By taking part in the study, participants in the usual care arm completed patient reported outcomes, received newsletters, and were potentially influenced to reflect on their LUTS, hence triggering health seeking behaviour that could improve their symptoms. The clinical importance of this result is potentially increased given that this pragmatic study of a non-drug intervention was unselective of type or severity of LUTS and was based in primary care. In addition, the result was sustained, with an interval (minimum nine months) between the end of input from a healthcare professional and measurement of the primary outcome.

We did not identify other studies of similar size directed at this issue. A non-randomised pilot study of men with uncomplicated LUTS in secondary care gave access to an online self-management programme in the intervention arm, versus usual care from a urologist.^22^ No significant differences was found between cohorts for the IPSS, and uptake of the intervention was only 53%. A randomised trial determined the effects of a health education strategy for older adults living at home, providing a booklet on five common health problems, including LUTS,^23^ and found that the health education strategy did not change visits to a doctor within three months. Both these studies suggest primary care is the most appropriate setting to support self-care in LUTS.

Post-void dribble affects about half of men,^24^ and incontinence affects about one man in eight.^25^ These are bothersome symptoms,^26^ so they were included in the standardised booklet used in the intervention. Neither symptom is captured by the IPSS, however, and therefore we used the International Consultation on Incontinence Questionnaire Urinary Incontinence-Short Form when men were assessed by healthcare professionals, to indicate who should be directed to the applicable sections of the booklet. At 12 months, the mean International Consultation on Incontinence Questionnaire Urinary Incontinence-Short Form score was 3.7 in the intervention arm and 4.5 in the usual care arm (out of a maximum score of 12). This small difference is unlikely to be clinically significant overall, but it does not exclude the possibility that individuals may have obtained a useful benefit. Similar benefits of likely low importance were observed for the IPSS quality of life (difference of 0.34 at 12 months) and possibly also men’s perception of their LUTS (difference of 4.78 at 12 months).

A strong focus on drug management of LUTS in men persists,^27^ perhaps encouraging clinicians to rely on drug use; however, men have tended previously to express a preference for conservative and less risky treatment for LUTS.^28^ The TRIUMPH study found that symptomatic improvement of LUTS can be sustained in the medium term using clear written materials. Key features were practical relevant assessment, interpretation by a suitably trained healthcare professional, focus on the most applicable elements for the individual’s symptoms, and supportive follow-up. The type of healthcare professional (nurse or healthcare assistant) undertaking the assessment and intervention did not appear to affect outcomes. Accordingly, the intervention appeared to be well suited to delivery by either type of healthcare professional in clinical practice.

### Strengths and limitations of this study

This was a large pragmatic randomised controlled trial conducted in a range of general practices in two English regions. Recruitment of practices and men was high, and delivery of the intervention was successful, including follow-up contacts to 12 weeks. Follow-up was timed to capture whether the impact of the intervention was sustained, with low missing data for patient reported outcomes at 12 months.

Some considerations are needed in interpreting the findings. The preference for conservative and less risky treatment for LUTS is potentially affected by baseline symptom severity,^28^ and the study randomised men to receive an intervention regardless of baseline severity, provided they considered their symptoms to be bothersome. The study could not distinguish which specific symptoms improved the most. Nocturia was included, but it can also have multifactorial bases driven by several medical influences,^19^ ^20^ and a qualitative exploration has been published finding that men who have long term disruptive symptoms, perceive that the booklet content was novel or worthwhile, and believe that self-management might help were more receptive to the intervention.^29^ The study was not able to distinguish which elements of the intervention are necessary for its success—for example, whether reduced follow-up contacts would have been sufficient. The predominance of participants of white ethnicity in the study populations may restrict applicability, particularly for other ethnic groups. This merits additional evaluation.

### Conclusions and future research

Guidelines recommend conservative management as the preferred treatment for LUTS in men. The TRIUMPH study showed that a standardised and manualised intervention achieved a sustained reduction in LUTS (difference in mean IPSS at 12 months of −1.81), which was less than the predefined target reduction of 2.0 points.

Future research will be directed at integrating the TRIUMPH intervention into general practice infrastructure, adapting it for patients with low literacy or whose first language is not English, including training materials, approaches to interpretation, and access to the standardised booklet. Potentially, many of the symptoms managed in this way are also experienced by women, raising the possibility of developing an equivalent standardised and manualised approach to managing LUTS in female patients.

**What is already known on this topic**

Assessment of LUTS in men and use of conservative treatments in primary care are limited and variable
Evidence that conservative treatments are effective for LUTS in men are limited, despite being recommended in national guidelines

**What this study adds**

This study developed a standardised and manualised intervention that provided a practical resource to support symptom assessment and conservative treatment for LUTS in men in primary care
The intervention achieved a sustained reduction in LUTS in a UK primary care setting (difference in mean International Prostate Symptom Score of −1.81 at 12 months), which was less than the predefined target reduction of 2.0 points

## Data Availability

All data requests should be submitted to the corresponding author for consideration. Access to anonymised data may be granted following review.

## Web extra

Extra material supplied by authors

Supplementary information: Additional tables S1-S3

Supplementary information: Administration checklist for study booklet

Supplementary information: Intervention booklet: Helping you control your waterworks

## Notes

Contributors: MJD was the chief investigator of TRIUMPH; he conceived the study, participated in its design and coordination, and drafted the manuscript. JAL, NC, MF, LM, HH, SJM, MJR, SN, JR, MJD, LAR, and GT assisted with the study design. JW and JF developed the trial procedures, protocol, and manuscript, and managed the coordination of the study. MM and JT assisted with the coordination of the study. SJM designed the statistical analysis, and ES conducted the statistical analysis. MC contributed to the data extraction from general practice medical records. All authors contributed to the oversight of the study via the Trial Management Group, read and commented on drafts of the manuscript, and approved the final version. MJD is the guarantor. The corresponding author attests that all listed authors meet authorship criteria and that no others meeting the criteria have been omitted.

The lead author (MJD) affirms that this manuscript is an honest, accurate, and transparent account of the trial being reported; that no important aspects of the trial have been omitted; and that any discrepancies from the trial as planned (and, if relevant, registered) have been explained.

Dissemination to participants and related patient and public communities: Participants of the TRIUMPH study will be informed of the results through its website (bristol.ac.uk/triumph-study) and will be sent details of the results in a study newsletter. Findings will also be disseminated through the media.

Provenance and peer review: Not commissioned; externally peer reviewed.
