# Supplementary material for: Treatment of lower urinary tract symptoms in men in primary care using a conservative intervention: cluster randomised controlled trial
Source: BMJ. 2023 Nov 15;383:e075219. doi: 10.1136/bmj-2023-075219 (PMC10646682; doi:10.1136/bmj-2023-075219)
Supplement: Supplementary file 1 — Supplementary information: Additional tables S1-S3 [file dram075219.wt1.pdf]

SUPPLEMENTARY TABLES

Table S1: Sensitivity analysis: Comparison of the results of ITT analysis with per protocol analyses

	N (int)*	N (usual care)*	Total	Mean	SD	Min - Max	Difference in means 95% CI	p-value
**ITT**	424	463	887	12.79	6.64	0 - 35	-1.81 (-2.66, -0.95)	<0.001
**Per protocol 1:** Including all in the usual care arm and those in the intervention who received the intervention booklet and had 3 follow-up contacts	391	463	854	12.84	6.67	0 - 35	-1.84 (-2.70, -0.99)	<0.001
**Per protocol 2**: Including all in the usual care arm and those in the intervention who received the intervention booklet and had 2 follow-up contacts	24	463	487	13.76	6.80	0 - 32	-0.83 (-3.16, 1.50)	0.485
**Per protocol 3**: Including all in the usual care arm and those in the intervention who received the intervention booklet and had 1 follow-up contact	5	463	468	13.88	6.85	0 - 32	-0.57 (-5.12, 3.99)	0.807
**Per protocol 4**: Including all in the usual care arm and those in the intervention who received the intervention booklet and had no follow-up contacts	2	463	465	13.90	6.83	0 - 32	-4.25 (-11.38, 2.89)	0.244
**Per protocol 5:** : Including all in the usual care arm and those in the intervention who received the intervention in the protocolised format (all 3 follow-ups, week 1 was delivered on the phone and week 4 or 12 delivered in the participants preferred follow-up method (not done face-to-face))	344	463	807	12.83	6.63	0-35	-2.06 (-2.92, -1.20)	<0.001

*Ns included in analysis (dependent on completion of IPSS at baseline and 12 month follow-up)

Table S2: Reasons for exclusion at GP manual screening

Reason for exclusion	**Patients excluded at GP manual screening;** n (% of all exclusions)
Currently being treated for prostate or bladder cancer	50 (2.4)
Lack of capacity	85 (4.2)
Patient does not have LUTS	871 (42.6)
Patient is under 18 years of age	4 (0.2)
Patient has poorly-controlled diabetes mellitus	18 (0.9)

Previous prostate surgery	71 (3.5)
Recently referred or currently under urological review	441 (21.5)
Relevant neurological disease or referral	56 (2.7)
Unable to complete assessments in English	9 (0.4)
Unable to pass urine without a catheter	61 (3.0)
Undergoing urological testing for LUTS	38 (1.9)
Visible haematuria	42 (2.1)
*Other**	301 (14.7)
	2,047

**Other: Awaiting surgery or had previous surgery (n=2), COPD (n=3), cancer (n=17), cognitive impairment (n=14), deceased (n=4), declined research (n=6), declined treatment for LUTS (n=4), under palliative care (n=6), family bereavement/illness (n=6), frail (n=39), housebound (n=19), may not be able to comply with follow-up (n=2), mental health or substance use issues (n=29), no symptoms (n=13), not living at home (n=17), no longer at practice (n=13), involved in another trial (n=50), permanent catheter (n=1), prostatitis (n=3), UTI (n=6), under bladder/bowel services (n=1), reason not given (n=9) and other (n=37).*

Table S3 Serious Adverse Events (identified through trial SAE reporting procedures)

	Received Intervention	Received Usual care	Overall
Adverse events			
Total number of SAEs; n	47	47	94
Total number of related AEs; n	0	0	0
Total number of deaths; n	2	1	3
Status of SAEs; n(%)[a]			
Resolved	45 (95.74%)	44 (93.62%)	89
Ongoing	0	2 (4.26%)	2
Died	2 (4.26%)	1 (2.13%)	3

[a] Denominator for the proportion is the total number of SAEs in treatment arm
