# Supplementary material for: Treatment of lower urinary tract symptoms in men in primary care using a conservative intervention: cluster randomised controlled trial
Source: BMJ. 2023 Nov 15;383:e075219. doi: 10.1136/bmj-2023-075219 (PMC10646682; doi:10.1136/bmj-2023-075219)
Supplement: Supplementary file 2 — Supplementary information: Administration checklist for study booklet [file dram075219.ww1.pdf]

Questionnaire/bladder diary scores			Drinks and water intake	Controlling bladder leakage	Controlling urgent need to urinate	Emptying bladder completely	Getting rid of the last drops	Reducing sleep disturbance	GP consultation
IPSS	**Q1 score** *Incomplete emptying*					Score of 3 or more ❑			
IPSS	**Q2 score** *Frequency*		Score of 3 or more ❑						
IPSS	**Q4 score** *Urgency*				Score of 3 or more ❑				
IPSS	**Q7 score** *Nocturia*		Score of 1 or more ❑					Score of 1 or more ❑	
ICIQ UI SF	**Q1 score** *How often leak*			Score of 1 or more ❑					
ICIQ UI SF	**Q4 part 2** *Leak before toilet*				Yes ❑				
ICIQ UI SF	**Q4 part 3** *Leak cough/sneeze*			Yes ❑					
ICIQ UI SF	**Q4 part 4** *Leak asleep*								Yes ❑
ICIQ UI SF	**Q4 part 5** *Leak active*			Yes ❑					
ICIQ UI SF	**Q4 part 6** *Leak after urinating*						Yes ❑		
ICIQ UI SF	**Q4 part 7** *Leak no reason*								Yes ❑
ICIQ UI SF	**Q4 part 8** *Leak all the time*								Yes ❑
Bladder diary *	**24 hour fluid intake**		2L or more ❑						
Bladder diary *	**No. of caffeine & alcoholic drinks daily**		More than 2 drinks ❑						
Bladder diary *	**No. times passes urine in the day**		8 or more times ❑						
Bladder diary *	**No. times passes urine at night**		1 or more times ❑					1 or more times ❑	
Bladder diary *	**Sensation score**			Score of 4 ❑	Score of 3-4 ❑				

* the "worst" complete 24 hour given, "Day"= between waking and sleeping, "Night"= between sleeping and waking

Participant ID ____________________
