# Supplementary material for: Treatment of lower urinary tract symptoms in men in primary care using a conservative intervention: cluster randomised controlled trial
Source: BMJ. 2023 Nov 15;383:e075219. doi: 10.1136/bmj-2023-075219 (PMC10646682; doi:10.1136/bmj-2023-075219)
Supplement: Supplementary file 3 — Supplementary information: Intervention booklet: Helping you control your waterworks [file dram075219.ww2.pdf]

Helping You Control Your Waterworks

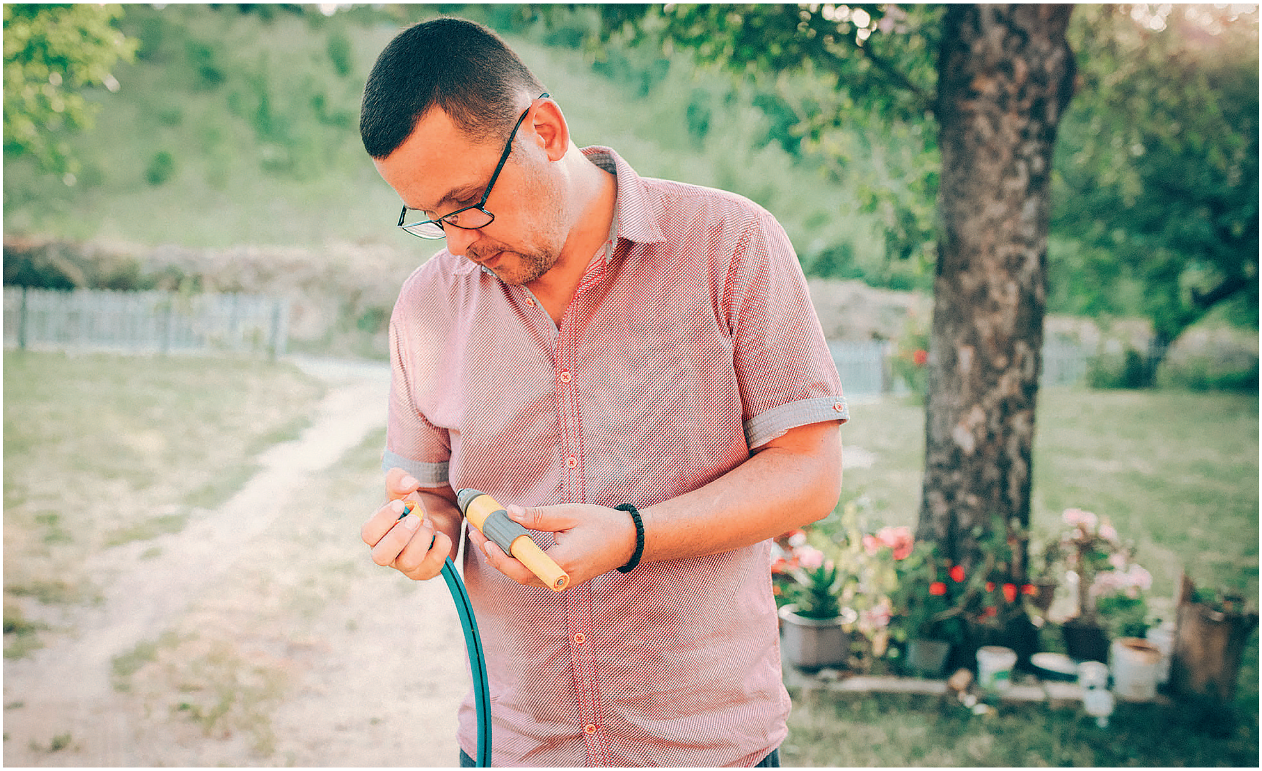

Guidance on urinary symptoms for men

About this booklet

Many men as they get older will experience changes in their urinary function, such as a slower urine stream, or changes in the frequency with which they pass urine. These and other symptoms which may occur are known as Lower Urinary Tract Symptoms, or ‘LUTS’.

The purpose of this booklet is to help men to understand these symptoms and to provide advice on how to manage them.

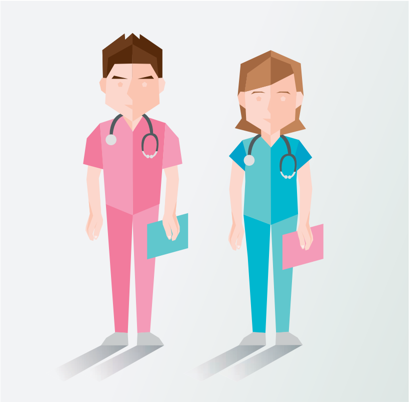

Your nurse is:

Name:……………………………

Email:………………………….…

Phone:…………………………...

If you have any queries or concerns regarding this booklet, your urinary symptoms or treatment, please speak to your nurse or GP.

‘Helping You Control Your Waterworks’ was written by expert doctors and nurses, working with the British Association of Urological Surgeons and with patients. It is part of the TRIUMPH study, funded by the National Institute for Health Research (NIHR) Health Technology Assessment (HTA) programme (ref 16/90/03).

© The University of Bristol 2018

Contents

Section	Page
Introduction	4
What is the urinary tract?	5
Urinary symptoms	7
Drinks and water intake	9
Controlling bladder leakage	17
Controlling an urgent need to pass urine	21
Emptying your bladder as completely as possible	23
Getting rid of the last drops	25
Reducing sleep disturbance caused by needing to pass urine	29
When you must see your doctor	33

© The University of Bristol 2018

Introduction

In this booklet we describe how the urinary tract works, different urinary symptoms, and what causes them. We also give you simple and sensible advice to help reduce urinary symptoms and their effect on your everyday life.

You will realise that some parts of the booklet apply to you more than others. We recommend you focus on the parts most relevant to you, as guided by your nurse or doctor. You are welcome to read the whole booklet if you are interested.

We hope that the information and advice will help reduce the impact of your urinary symptoms. It was written by experts using the latest medical evidence. However, if you feel that the improvement is not enough, and you are still bothered by your symptoms, contact your nurse or doctor to discuss further medical assessment.

This booklet is for men whose doctor has not indicated a serious medical problem is present.

If you experience certain symptoms described on page 33 you must contact your nurse or doctor.

© The University of Bristol 2018

1.What is the urinary tract?

The organs responsible for making, moving and storing urine are known as the urinary tract. Urine is made in the kidneys to get rid of waste products and excess water or salt from the blood. Urine passes from the kidneys down into the bladder. The bladder is a storage container for urine – most men can hold a pint of urine (about half a litre) in their bladder before having to pass urine, but it does vary a lot.

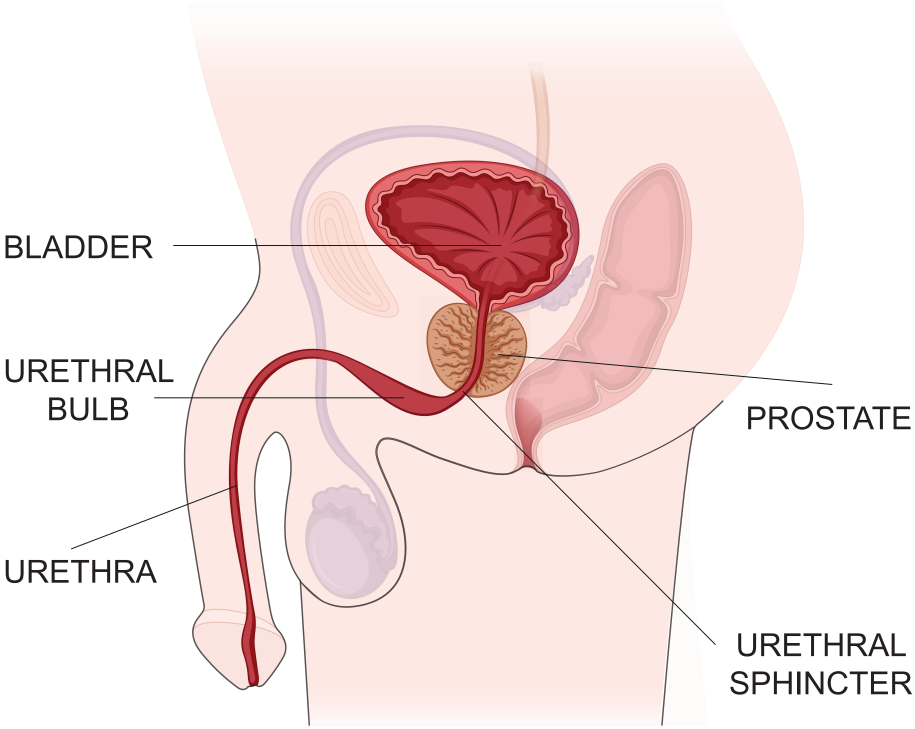

© The University of Bristol 2018

As your bladder fills, you will start to feel the need to pass urine, and you will go to the toilet. This might be because your bladder feels full. Or you may be about to do something where toilet breaks would be inconvenient (for example, a long journey, a theatre trip, or when going to bed).

When you are ready to pass urine two things happen:

1. Your bladder squeezes down (like a deflating balloon) to push out all the urine
2. A muscle at the exit of the bladder called the ‘urinary sphincter’ opens

Urine then passes from your body through the urethra, which is a tube that runs from your bladder to the tip of your penis. The urethra also passes through the prostate gland, a sexual organ which receives sperm from the testicles and makes semen.

© The University of Bristol 2018

2.Urinary symptoms

If your urinary system does not work as it should, you will experience what are described by doctors as 'Lower Urinary Tract Symptoms', or LUTS. Men of any age can get LUTS, but they are more common as you get older. Many men become aware of mild LUTS in their 40s. LUTS only really need treatment if they significantly affect your everyday life, since it is rare that they are linked to a serious health problem.

Symptoms while trying to pass urine

Symptoms that restrict your ability to pass urine are known by doctors as 'voiding LUTS'. Voiding LUTS can be a result of your prostate gland enlarging and pressing on your urethra. Very similar symptoms happen if your bladder loses its ability to squeeze down for emptying ('underactive').

Symptoms at other times

You can also experience symptoms when you are not intentionally passing urine, which are called 'storage LUTS'. These can happen if the kidneys make unusually large amounts of urine, the bladder muscle becomes overactive, or the bladder gets irritated.

You may experience either storage and / or voiding LUTS - it is common for men to have both types.

© The University of Bristol 2018

Types of LUTS and where to find advice

The various types of LUTS are listed in the following table with where to find advice in this booklet.

<table>
<tr><th>Urinary Symptom</th><th>Definition</th><th>Page</th></tr>
<tr><td>Increased urinary frequency</td><td>Need to pass urine often</td><td>9-16</td></tr>
<tr><td>Urgency</td><td>Need to pass urine without much warning</td><td rowspan="2">9-16
17-20
21-22</td></tr>
<tr><td>Urgency incontinence</td><td>Urgency resulting in leakage of urine which you cannot control</td></tr>
<tr><td>Polyuria</td><td>Passing unusually large amounts of urine</td><td>9-16</td></tr>
<tr><td>Nocturia</td><td>Being woken from sleep by the need to pass urine</td><td>9-16
29-32</td></tr>
<tr><td>Post voiding dribble</td><td>A small leak of urine straight after finishing urinating</td><td>25-28</td></tr>
<tr><td>Sensation of incomplete emptying</td><td>A feeling that there is still urine in your bladder even though you recently passed urine</td><td>23-24</td></tr>
</table>

© The University of Bristol 2018

3. Drinks and water intake

Your body can only store a certain amount of water or salt. If you have too much of either, your body will get rid of the excess in your urine, leading to an increase in the amount of urine you produce. Doctors call overproduction of urine 'polyuria'. This will mean you go to the toilet frequently and generally pass large volumes of urine each time.

For younger people, the body usually gets rid of excess water and salt while you are awake. However, as you get older your body changes and the increased urination may start to wake you up overnight. Waking in the night because you need to urinate is referred to by doctors as 'nocturia'.

Some medicines called 'diuretics' (for example, furosemide) are designed to increase the amount of urine you make, to help get rid of water or salt. Some types of drink can have a similar 'diuretic' effect, especially those containing caffeine or alcohol.

If you keep passing large amounts of urine, it could be because your diet has too much salt, water, caffeine or alcohol.

© The University of Bristol 2018

Your thirst is your body's guide to how much water you need. However, there are a small number of people who have a medical condition responsible for the body losing water excessively. These people will feel thirsty a lot of the time, even though they are drinking a lot. If this applies to you, please talk to your GP.

Advice on drinks and water intake

If you have polyuria, urinary frequency, urgency or nocturia (see the table on page 8), you should consider the following advice regarding drinks and food:

a) Keep your salt intake down

Avoid sprinkling salt on your food, avoid processed meals, and avoid artificial fizzy drinks.

Some common processed foods and take away foods can contain a lot of salt.

© The University of Bristol 2018

Processed foods come in packets which often tell you how much salt is present.

Here are three examples. On the left, the packet tells you a portion has 3% of your recommended daily intake; try to stick to foods which have less salt than this. Some packets use a traffic light system. A green label is good, as shown in the middle. A red label for salt would not be helpful for your urinary symptoms and should be avoided, as shown on the right.

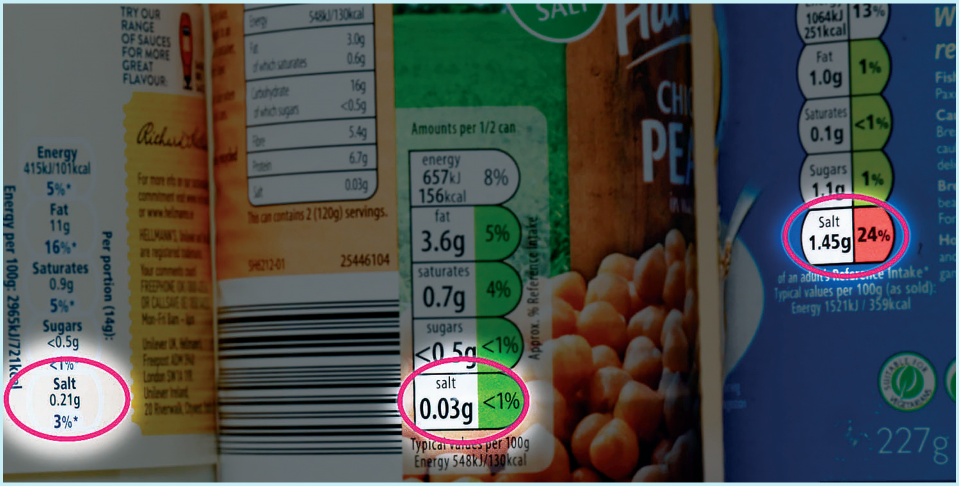

The NHS also provide guidance on their website: www.nhs.uk/live-well/eat-well/salt-nutrition

© The University of Bristol 2018

If you have previously had specific advice from your doctor on your salt intake, for example because your blood pressure is unusually low, please contact your doctor before following this advice.

b) Keep your caffeine intake down

Try to reduce your intake of coffee, tea and fizzy drinks containing caffeine.

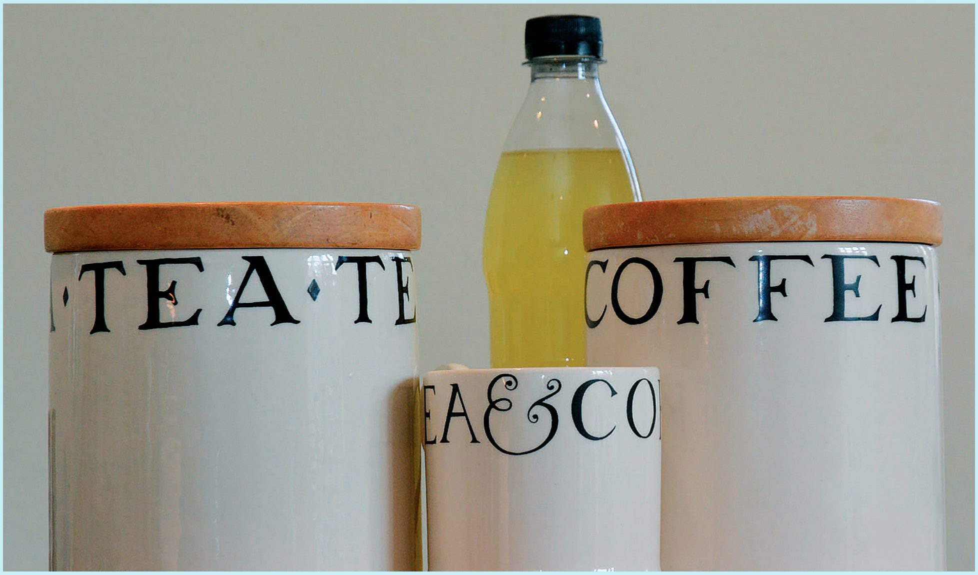

Avoid consuming a lot of drinks containing caffeine, such as coffee, tea, green tea and some fizzy drinks (especially energy drinks). Check the label if you are unsure.

© The University of Bristol 2018

When you first start to reduce these, you may get some cravings, but the cravings will ease off over a couple of days. You may find it easiest to reduce your daily intake of these drinks steadily over a couple of weeks.

c) Drink when you are thirsty

How thirsty you are should tell you whether you need to have a drink. If you are not thirsty, don't deliberately drink extra water.

d) Drink a sensible amount of fluid

The equivalent of roughly ***three pints daily*** (one and a half litres) is a healthy fluid intake for most people. You should drink at least two pints of fluid each day, and not deliberately dehydrate yourself as a way of managing urinary symptoms.

© The University of Bristol 2018

An example of your daily 3 pints of fluid:

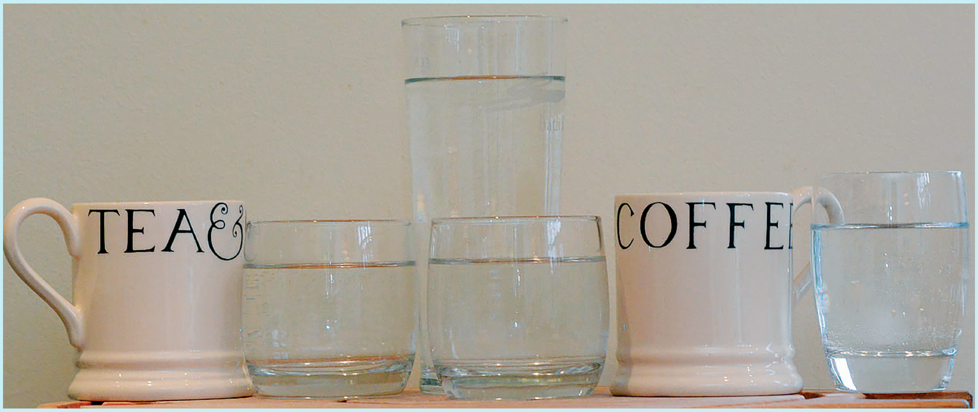

This amount covers all your drinks including water, tea, coffee and any other drinks consumed. However, ideally the ***main thing you drink should be water.***

You will need to drink more if you lose a lot of water for some reason (for example, on a hot day, doing sweaty exercise, becoming unwell, or getting diarrhoea). If your food contains a lot of water (for example salads, vegetables or pasta), you may not need to drink quite so much.

© The University of Bristol 2018

These foods, and other similar foods, contain a lot of water when prepared and served.

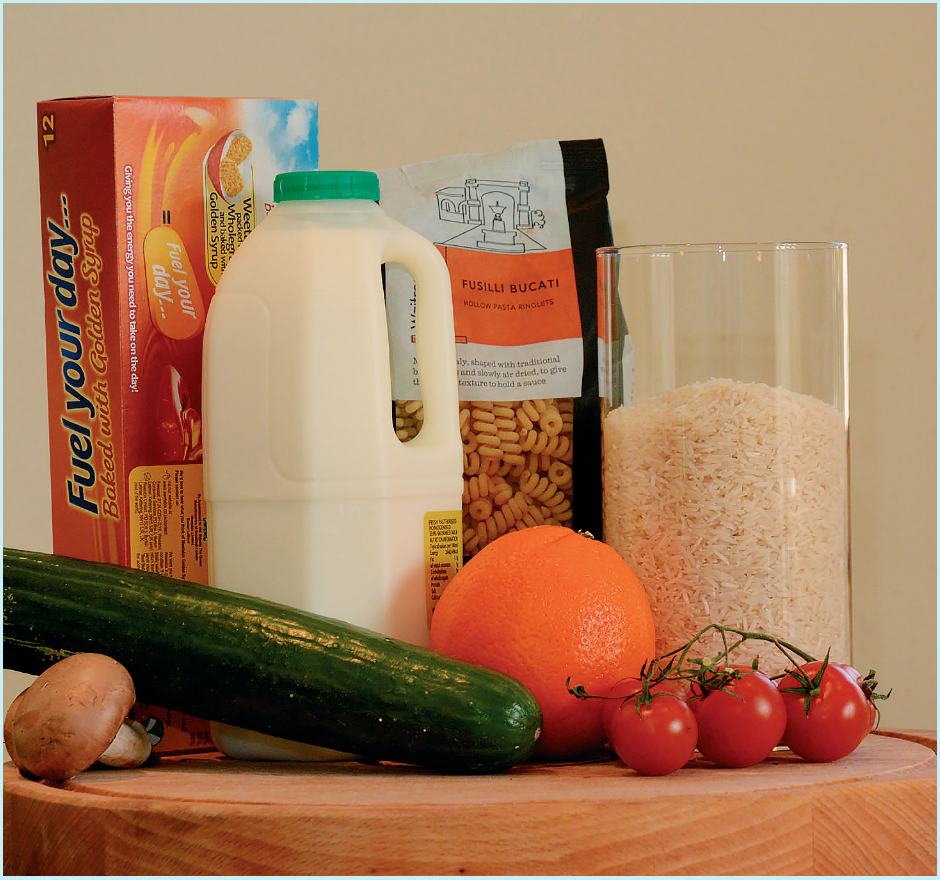

© The University of Bristol 2018

e) Only drink a sensible amount of alcohol

This is particularly important. A healthy intake of alcohol is less than 14 units in total during a whole week from all alcoholic drinks.

Example of maximum alcohol intake for a week:

Bottle of wine: *9 units*
Larger bottle of beer: *2.1 units*
Smaller bottle of beer: *1.3 units*

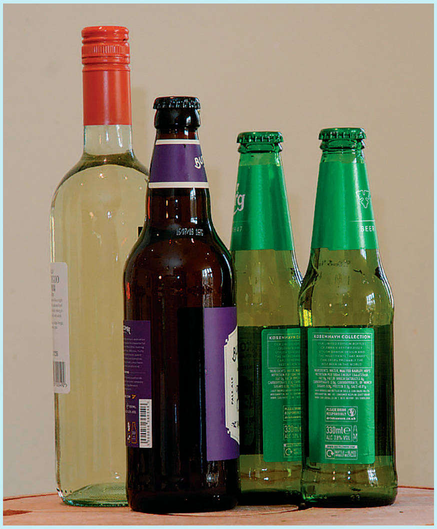

Units of alcohol will vary depending on the strength.
The number of units of alcohol is usually written on the packaging (but may be small and difficult to see).

Advice on alcohol intake is available from the NHS: www.nhs.uk/live-well/alcohol-support/calculating-alcohol-units and Drinkaware: www.drinkaware.co.uk

If you successfully follow this advice, you could notice your urinary symptoms reduce over a period of 1 to 4 weeks.

© The University of Bristol 2018

4. Controlling bladder leakage

Your pelvic floor muscles stretch from your pubic bone at the front, between your legs to the bottom of your spine. They work to support your bladder and bowel, and your body uses them to control when you urinate and empty your bowels. Doing pelvic floor muscle exercises to strengthen your pelvic floor may help you control urinary urgency.

Following the exercises on the next page will teach you how to tighten your pelvic floor muscles quickly and strongly. This will help you to resist an urgent need to pass urine.

Tightening your pelvic floor muscles strongly after you urinate can also be helpful if you experience dribbling of urine after you have finished passing urine.

© The University of Bristol 2018

How to do pelvic floor muscle exercises: Identifying the correct muscles

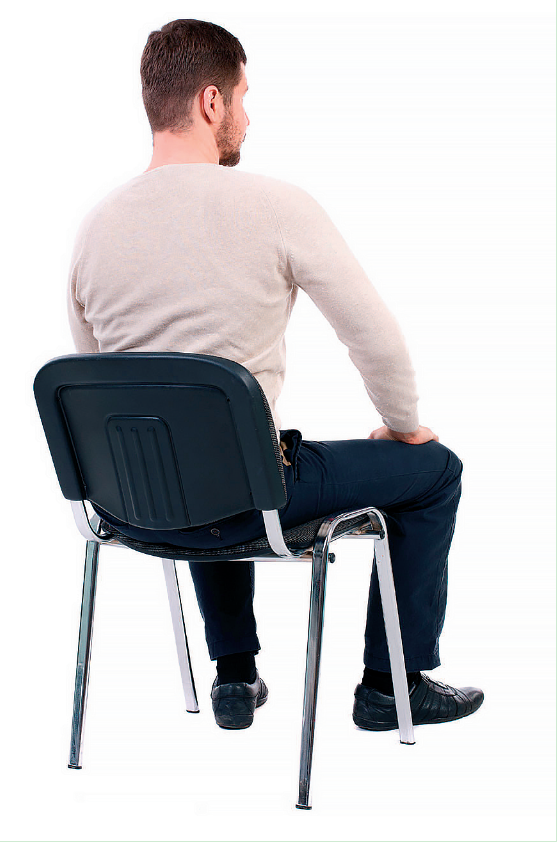

To make sure you use the right muscles, sit on a comfortable chair, leaning slightly forward with your feet slightly apart on the floor.

Try to practice two things:

1. Tighten the muscles you use to clench your anus, as if you're trying to control wind. Do this for a few seconds and then relax.
2. Also tighten the muscles you would use if you had to interrupt the flow whilst passing urine. Do this for a few seconds and then relax. *E.g. imagine someone comes around the corner when you are urinating.*

© The University of Bristol 2018

- Make sure your buttocks, thighs and abdomen stay relaxed throughout, and just breathe normally.
- Once you can do this sitting down, try to use the same muscles when standing up or lying down.
- If you feel the back of your scrotum move downwards and the anus bulge out slightly then you are doing it wrong.
- **When you use the right muscles, you can feel the back of your scrotum move upwards**

Once you feel confident you're using the correct muscles, you can begin to make the pelvic floor stronger, which means you need to do regular exercises.

You should do some **strengthening exercises**, so you can resist urinary urgency. You should also do some **speed exercises**, so you can use your pelvic floor quickly when needed.

© The University of Bristol 2018

Pelvic floor exercises: how many and how often?

Strength exercises: Slowly tighten the muscles as hard as you can. Try to hold this lift for 10 seconds. Don't worry if you can't manage that long to begin with, how long you can hold will improve over time. Slowly relax the muscles and rest for 10 seconds. Aim to repeat the lift and rest up to 10 times.

Speed exercises: Do the same action but tighten the muscles as quickly as possible. Hold for one second and then let go. Do 10 of these short, fast lifts.

You should aim to do a set each of the slow and fast exercises at least three times a day to build up strength and speed.

If you do this regularly, you should notice your pelvic floor is stronger after a couple of months. Once it is strong, doing a set of both type of exercises twice a day should help maintain what you've achieved.

© The University of Bristol 2018

5. Controlling an urgent need to pass urine

Urgency is described by doctors as a sudden desire to pass urine which is difficult to defer. Patients often use the phrase 'when I've got to go, I've got to go'. Men with urinary urgency may worry that they could leak, and so they rush to the toilet.

If you experience urinary urgency, you should do three things:

1. Follow the advice on **drinks and fluid intake** given on pages 9-16.
2. Use a technique called **'bladder training'**, described in this section.
3. Make sure you have read the information on pages 17-20, because you need to know how to contract your pelvic floor muscles effectively to do bladder training.

© The University of Bristol 2018

Bladder Training

When you get the urgency symptom, try to resist passing urine by using your pelvic floor muscles: to begin with, contract your pelvic floor for a few seconds (in the early days, just count to 5). Only when you have counted to 5 should you then head for the toilet.

You can also try to distract yourself from the urgency feeling. For example, through tasks that involve mental concentration such as crossword puzzles, or using self-motivational statements such as ‘I can wait’.

As you get stronger and more confident, you can increase the time between the onset of urgency and heading to the toilet. Increase the time a little bit more each week, according to whatever you feel you can comfortably manage. Until you have greater control, you may feel more confident if you use continence pads (e.g. Tena™ for men). These can be purchased from supermarkets and pharmacies.

After about a month, you may feel able to leave it a full minute before going to the toilet, and in that time the urgency may even pass away altogether.

© The University of Bristol 2018

6. Emptying your bladder as completely as possible

It is quite natural to have a small amount left in your bladder after passing urine, and it does not generally cause any problems. However, some people can have a distinct feeling that there is a significant amount still in their bladder and find this feeling a nuisance.

It's worth remembering that research has shown that people are often inaccurate when they estimate what's in their bladder. So, a feeling that there is a significant amount still in your bladder may not be correct. Try to distract yourself from the feeling that your bladder has not emptied fully.

The key to emptying your bladder as well as you possibly can is to take your time. This is most crucial in a public toilet and you may feel more comfortable using the cubicle to start with. This means you can sit down on the toilet, which will make spraying less of a problem. You still need to take your time, even if you become aware that someone who arrived after you has finished before you. When you think about it, no-one is especially interested in someone else's toilet speed.

© The University of Bristol 2018

Advice on emptying your bladder as completely as possible

To give yourself the best chance of fully emptying your bladder when you pass urine, try to avoid being in a rush, or feeling pressured to get on with it. It's important to **relax and take as long as you need**.

Stay by the toilet or urinal for an extra 20 seconds, staying relaxed and focussed on passing urine.
Then you may manage to get rid of some more urine. Doctors may refer to this as 'double voiding'.

Pushing urine out by straining may help, as you might do when you empty your bowels, but just for a couple of breaths. If straining does not seem to achieve anything, don't keep trying it.

© The University of Bristol 2018

7. Getting rid of the last drops

Many men complain about a dribble of urine escaping into their underwear just after they've been to the toilet to pass urine. Doctors call this a 'post voiding dribble', and it can cause a patch the size of a 50p coin in the underwear, or even some visible staining of the trousers. It can affect men of any age, but the most common age to become aware of this problem is in your 40s.

To understand the problem, you need to know where your urethra is, which is the tube running from your bladder to the tip of your penis. Have a look at the picture on page 5, which shows the important parts. As your urethra leaves your bladder, it heads downwards to a point in front of your anus. The urethra then heads forwards into its widest part, the urethral bulb. From there the urethra passes underneath the pubic bone, and above your scrotum, where it runs along the length of your penis.

After you have passed urine, some can remain in the urethral bulb. Shaking the penis does not get rid of it. The urethral bulb is about the size of your thumb, and rather squashy. If you are not sure where it is, feel the area next time you get an erection, as this makes the urethral bulb become more prominent.

© The University of Bristol 2018

Getting rid of those last drops trapped in the urethral bulb

Learning the skill is easiest when sitting down.

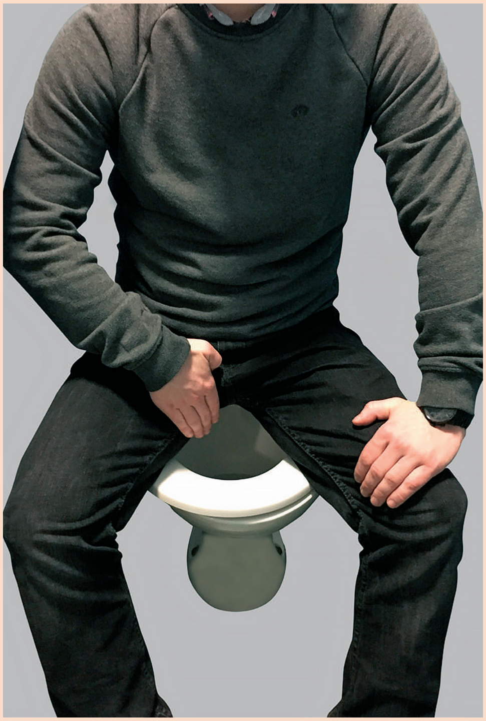

- Sit down on the toilet (trousers and pants down by your ankles) with your knees slightly apart
- Reach behind the scrotum with your middle and ring fingers
- Press upwards an inch in front of the anus to squeeze the back end of the urethral bulb
- Keeping the upwards pressure, slide your fingers forwards towards your penis
- Press firmly but without causing discomfort
- When your fingers have reached the underside of your penis, you can put your thumb on top of the penis to push the drops all the way out

© The University of Bristol 2018

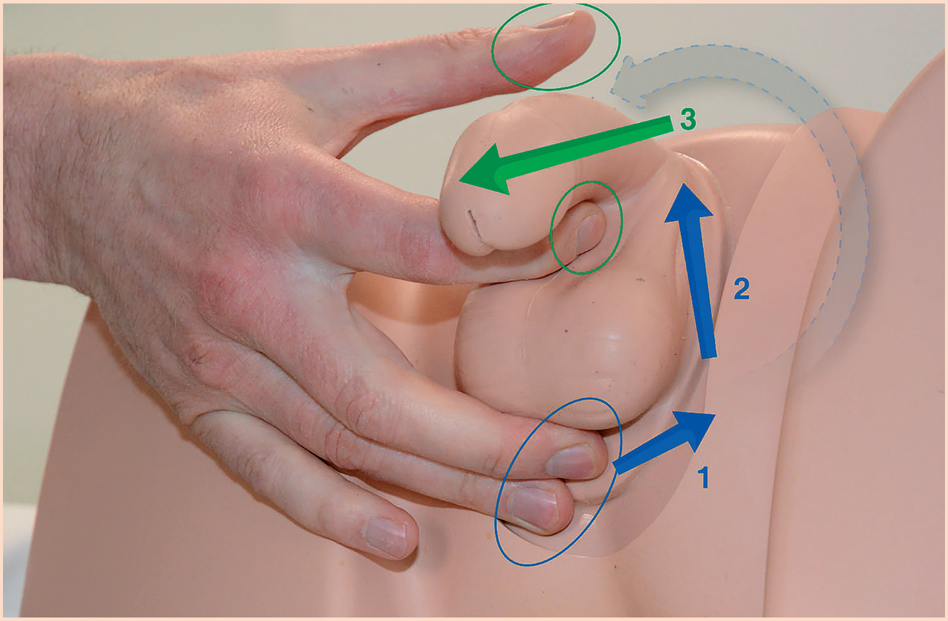

Pressing out the last drops from the urethral bulb:

1. *Press upwards gently but firmly, between the anus and scrotum*
2. *Gently keeping the pressure on, ease your fingers forwards, so the urine can be eased forwards towards the penis*
3. *You can shake the last drops from the penis*

The knack is to make sure you keep the pressure on when you reach the scrotum, because your testicles can get in the way. Doing this takes some practice to avoid discomfort. It is important not to let go, because the drops you're trying to get rid of will probably go back to the urethral bulb.

© The University of Bristol 2018

Once you've mastered it sitting down, you can start to try the technique standing up. Stand with your legs shoulder-width apart, keep your legs straight and push your bottom out. Press upwards from anus to scrotum as described on the previous page. To begin with, have your trousers and pants right off.

Once you've really mastered it, you will realise you can do this in just a few seconds. You will also be able to try it with your trousers on, provided the trouser crotch is not too tight (i.e. slacks, rather than jeans).

Getting skilful enough to get rid of the last drops quickly, whilst standing up with your trousers on, makes it possible to do it in public toilets discretely. Understanding and following this advice on getting rid of the last drops can take about 8 weeks to get skilful.

© The University of Bristol 2018

8. Reducing sleep disturbance caused by needing to pass urine (nocturia)

If you don't get a good night's sleep, it affects you badly on the following day, particularly if it happens often. Most of the time, people can cope if their night's sleep is only interrupted once. Waking up twice or more often is the point at which people really begin to notice being deprived of sleep.

Several things can cause sleep disruption:

- Not relaxing as you approach bedtime, for example working or answering emails till late in the evening, or focussing on a small phone screen
- A feeling of anxiety
- An environment that is not well set-up for sleep, e.g. having a noisy or bright bedroom
- Using stimulants in the evening, such as caffeine, alcohol or some drugs

Waking up because of a need to pass urine is known by the medical term of 'nocturia'. This is not the same as waking up for some other reason, and then deciding to pass urine. Nocturia is a complex symptom which may be caused by natural changes in your body and some medical conditions. Nocturia is a common cause of sleep disruption, and it is something men start to notice happening more often as they get older.

© The University of Bristol 2018

To reduce sleep disturbance

Think about your sleeping arrangements and toilet facilities, to see whether any adjustments can help:

- Can you reduce noise in your bedroom? If not, should you use earplugs?
- Can you reduce brightness? If not, should you use an eye mask?
- Is your bedroom a suitable temperature? The NHS advises a temperature of between 18°C and 24°C to aid sleep
- Are your bedclothes so warm that you get too hot?
- To reduce disturbing your partner, can you adjust things to make it easier to get to the toilet quickly and quietly? You may be able to oil a squeaky hinge, or use a torch if the light switch is noisy. Consider not flushing the toilet until the morning.

© The University of Bristol 2018

To reduce the effect of nocturia on you, there are some things you can control which might help:

Get into good habits to help yourself nod off…
a) Take some exercise every day; this helps you to feel tired at bedtime, and also helps your body get rid of surplus water. The NHS provides guidance on exercise on their website:
www.nhs.uk/live-well/exercise

b) Don't eat too late, and most importantly don't have a large fatty meal late in the evening.

c) Make time to do something pleasant and relaxing for at least half an hour before bedtime.

d) Do not look at emails just before turning out the light, or later on in the night.

In the evening, avoid things that increase urine output…

e) You need to follow the advice on drinks and liquid intake given on pages 9-16, taking care to avoid unnecessary liquid in the few hours before bedtime.

© The University of Bristol 2018

f) Avoid consuming stimulants in the evening, for example coffee, tea, and fizzy drinks with artificial sweeteners or flavourings.

g) Avoid alcoholic drinks. As well as containing a lot of water, the alcohol has a stimulating effect which makes it harder to get back to sleep once you've been woken up. There are several health benefits of ensuring your alcohol intake is sensible, described in the NHS advice available at: www.nhs.uk/live-well/alcohol-support/the-risks-of-drinking-too-much

When you do wake up during the night…

h) Work on relaxation techniques, to help you get back to sleep. The NHS recommend 'Don't lie in bed feeling anxious about lack of sleep. Instead, get up, go to another room for about 20 minutes and do something else, such as reading or listening to soft music, before trying again.'

Following this advice should help in about 4 to 8 weeks. It is unlikely to stop you needing the toilet overnight altogether, but hopefully will lead to fewer episodes, and reduce their impact.

© The University of Bristol 2018

9. When you must see your doctor

This booklet is for advice only. The booklet does not replace any specific tests or treatments your doctor has arranged for you.

It is important to let your doctor know if things change substantially after you were assessed. Certain symptoms could suggest you have developed a serious problem. For example, you must see your doctor if:

- You see blood in your urine
- You find it really difficult to pass urine and develop a swollen bladder
- You start wetting the bed while asleep
- You have a constant thirsty feeling
- You lose feeling around your anus and genitals
- You start to leak urine (incontinence); this does not include post voiding dribble (see page 25).

© The University of Bristol 2018

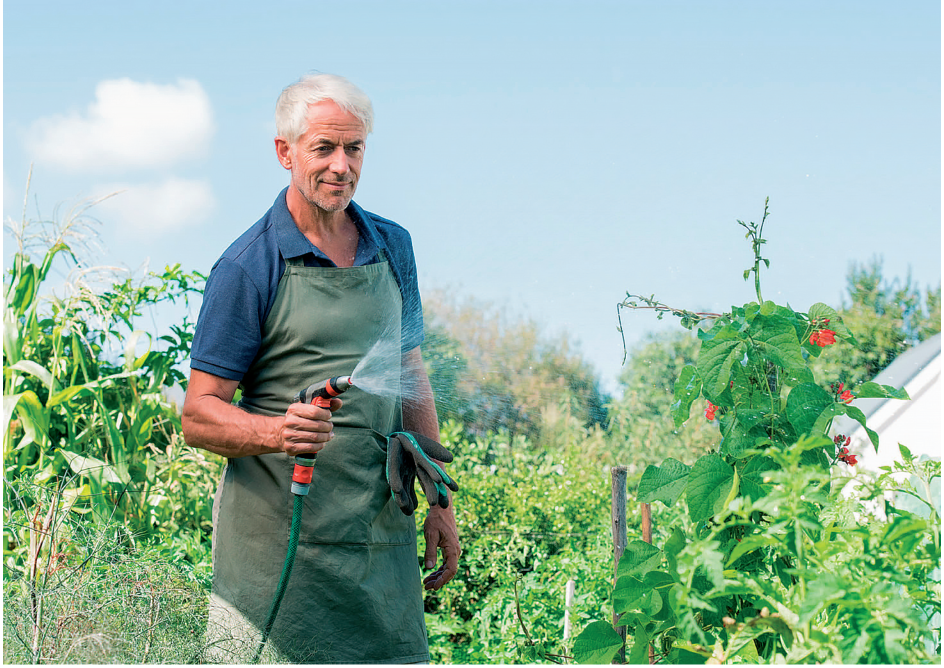

TRIUMPH

University of BRISTOL

UNIVERSITY OF Southampton

NHS
Bristol, North Somerset
and South Gloucestershire
Clinical Commissioning Group

Bristol Randomised Trials Collaboration

Funded by
NHS
National Institute for
Health Research

The views expressed are those of the author(s) and not necessarily those of the NHS, the NIHR or the Department of Health and Social Care.

© The University of Bristol 2018
